# Quantification of NAD(P)H in cyanobacterial cells by a phenol extraction method

**DOI:** 10.1007/s11120-021-00835-1

**Published:** 2021-05-02

**Authors:** Kenya Tanaka, Ginga Shimakawa, Hiro Tabata, Shoko Kusama, Chikahiro Miyake, Shuji Nakanishi

**Affiliations:** 1 Graduate School of Engineering Science, Osaka University, 1-3 Machikaneyama, Toyonaka, Osaka 560-8631 Japan; 2 Research Center for Solar Energy Chemistry, Osaka University, 1-3 Machikaneyama, Toyonaka, Osaka 560-8631 Japan; 3 Department of Biological and Environmental Science, Faculty of Agriculture, Graduate School of Agricultural Science, Kobe University, 1-1 Rokkodai, Nada, Kobe, 657-8501 Japan

**Keywords:** NADPH, Photosynthesis, Cyanobacteria, Quantitative determination, Intracellular redox state

## Abstract

In photosynthetic organisms, it is recognized that the intracellular redox ratio of NADPH is regulated within an appropriate range for the cooperative function of a wide variety of physiological processes. However, despite its importance, there is large variability in the values of the NADPH fraction [NADPH/(NADPH + NADP^+^)] quantitatively estimated to date. In the present study, the light response of the NADPH fraction was investigated by applying a novel NADP(H) extraction method using phenol / chloroform / isoamyl alcohol (PCI) in the cyanobacterium *Synechocystis* sp. PCC 6803. The light response of NADP(H) observed using PCI extraction was qualitatively consistent with the NAD(P)H fluorescence time course measured in vivo. Moreover, the results obtained by PCI extraction and the fluorescence-based methods were also consistent in a mutant lacking the ability to oxidize NAD(P)H in the respiratory chain, and exhibiting a unique NADPH light response. These observations indicate that the PCI extraction method allowed quantitative determination of NADP(H) redox. Notably, the PCI extraction method showed that not all NADP(H) was oxidized or reduced by light–dark transition. Specifically, the fraction of NADPH was 42% in the dark-adapted cell, and saturated at 68% in light conditions.

**Supplementary Information:**

The online version contains supplementary material available at 10.1007/s11120-021-00835-1.

## Introduction

The redox pair NADP^+^/NADPH is involved in various reactions in photosynthetic organisms. NADP^+^ is the terminal electron acceptor in the photosynthetic electron transport chain (PETC) and is converted to NADPH under light conditions. The NADPH thus generated supports biosynthetic and antioxidant systems by serving as a reducing driver for various enzymes including glyceraldehyde 3-phosphate dehydrogenase (GAPDH), NADPH-thioredoxin reductase (NTR), glutathione reductase (GR), etc. (Raines 2003; Hishiya et al. 2008; Yoshida and Hisabori 2016; Vogelsang and Dietz 2020). The oxidized form (NADP^+^) also functions as an essential co-factor for glucose-6-phosphate dehydrogenase (G6PDH), 6-phosphogluconate dehydrogenase (6PGDH), and isocitrate dehydrogenase (ICD) in primary metabolism (Muro-Pastor and Florencio 1992; Ishikawa and Kawai-Yamada 2019). Given that the NADP^+^/NADPH redox ratio, a critical factor influencing various biological processes, can vary with changes in the lighting conditions, it is vitally important to quantitatively determine the intracellular concentrations of NADP(H) for a deeper understanding of the physiology of photosynthesis.

Fluorescence detection of NADPH is a representative in vivo method for measuring light-responsive changes in NADPH concentrations. For example, it has been shown that NADPH was produced or consumed in the sub-second order by light–dark transitions (Mi et al. 2000; Kauny and Sétif 2014; Shaku et al. 2016). However, the fluorescent yield of NADPH changes depending on the peripheral environment (Latouche et al. 2000; Kauny and Sétif 2014). Importantly, NADP^+^ (the counterpart of NADPH) does not emit fluorescence. Therefore, the in vivo fluorescence-based technique cannot directly assess the NADP^+^/NADPH redox ratio or the absolute amount of NADP(H). In contrast, the concentration of NADP(H) can be quantitatively measured following cellular extraction. In fact, previous studies have attempted to quantify the concentration of NADP(H) and the redox ratio in vitro. However, the reported values of the in vitro studies differ widely (Table 1). The redox ratio estimated by in vitro extraction methods must be consistent with the dynamic behavior of NADPH fluorescence; however, no studies have been undertaken to demonstrate consistency between in vivo and in vitro measures.

**Table 1:** Comparison of NADP(H) measurements in cyanobacteria and their results

Species	NADPH^3^	NADP^+^^3^	NADPH fraction	Growth condition	Extracted condition	Extraction method	References
*Synechococcus*^1^	373 μM	276 μM	0.575	6 h Light		Acid/Base	Tamoi et al. (2005)
*Synechococcus*^1^	306 μM	177 μM	0.634	6 h Dark		Acid/Base	Tamoi et al. (2005)
*Synechocystis*^2^	4.8 μmol/mg Chl	1.6 μmol/mg Chl	0.750	Photoautotrophic		Beads	Cooley and Vermaas (2001)
*Synechocystis*^2^	138 nmol/g FW	99.6 nmol/g FW	0.581	Photoautotrophic		Organic solvent	Takahashi et al. (2008)
*Synechocystis*^2^	88.0 nmol/g FW	70.3 nmol/g FW	0.556	Photomixotrophic		Organic solvent	Takahashi et al. (2008)
*Synechocystis*^2^	43.6 nM	6260.8 nM	0.0069	Photoautotrophic		Organic solvent	Osanai et al. (2014)
*Synechocystis*^2^	58.8 nmol/g FW	69.0 nmol/g FW	0.460	Photoautotrophic		Acid/Base	Ishikawa et al. (2016)
*Synechocystis*^2^	17.9 nmol/g FW	65.9 nmol/g FW	0.214	Photoautotrophic		Acid/Base	Ishikawa et al. (2019)
	59.9 nM/OD	82.0 nM/OD	0.422		Dark-adapted		This study
*Synechocystis*^2^	87.4 nM/OD	42.0 nM/OD	0.677	Photoautotrophic	Light-irradiated	PCI	This study
	45.3 nM/OD	98.9 nM/OD	0.315		Onset of darkness		This study

^1^*Synechococcus elongatus* PCC 7942

^2^*Synechocystis* sp. PCC 6803

^3^For amount values of NADP(H) in this study, unit can be converted by following Eqs. 1 nM/OD = 0.25 nmol/mg Chl (using value of 4.0 mg Chl/L/OD from Fig. S1)

In the present work, we quantified the amount of NADP(H) in *Synechocystis* sp. PCC 6803 (hereafter *Synechocystis*) in vitro using a phenol/chloroform/isoamyl alcohol (PCI) solution to deactivate undesirable enzymatic reactions, and evaluated the obtained values against the dynamic behavior of NAD(P)H fluorescence. Importantly, the light-responsive changes in NADP(H) determined using the PCI extraction method were consistent with the results of in vivo NAD(P)H fluorescence measurements, indicating the validity of the novel extraction protocol. Development of this novel protocol has revealed that the fraction of NADPH [NADPH / (NADPH + NADP^+^)] in *Synechocystis* cells in light conditions reached 68%, which was unchanged over wide range of light intensity.

## Materials and methods

### Bacterial strains and cell culture conditions

We used the following *Synechocystis* strains: wild-type and *ΔndhD1/2* (Ohkawa et al. 2000). These strains were grown and maintained on solid (1.5% Bacto agar) BG-11 medium plates. For pre-culture, cells from the agar plate were inoculated into 30 mL liquid BG-11 medium in a 100-mL flask and grown at 30 °C with air bubbling under white light illumination at an intensity of 20 µmol m^−2^ s^−1^. For the main culture, the pre-culture was inoculated to achieve an optical density of 0.02 at 730 nm (OD_730_) in 30 mL BG-11. Other conditions were the same as those in pre-culture. Fig. S1 shows the growth curves of the main culture measured at an optical density of 730 nm and the chlorophyll concentration. To determine the chlorophyll concentration, the cells were harvested by centrifugation at 12,000 × g for 5 min and suspended in 100% methanol. The suspension was then centrifuged at 12,000 × g for 5 min, followed by measurement of absorption at 665 nm. The chlorophyll *a* concentration was calculated according to a previously described method (Grimme and Boardman 1972).

### NAD(P)H fluorescence measurements

The in vivo NAD(P)H fluorescence originating from NAD(P)H was measured using the NADPH/9-AA module of a Dual-PAM-100 instrument (Heinz Walz, Effeltrich, Germany) (Kauny and Sétif 2014; Shimakawa et al. 2018). The reaction mixtures (2 mL) contained fresh BG-11 medium (pH 7.5) and cyanobacterial cells (2.5 μg chlorophyll mL^−1^). The NADPH/9-AA module consists of an emitter unit (DUAL-ENADPH) and a detector unit (DUAL-DNADPH). NADPH fluorescence was excited by UV-A (365 nm) from the DUAL-ENADPH unit and detected by a blue-sensitive photomultiplier with a filter transmitting light between 420 and 580 nm in the DUAL-DNADPH unit. The measured light intensity was on a scale from 1 to 20, and the intensity was set at 10 in this study. The measuring light frequency in the absence and presence of red actinic light was set at 200 Hz and 5,000 Hz, respectively.

### Pyridine nucleotides extraction

PCI solution was prepared as follows. Crystalline phenol was melted in a 65 °C water bath, followed by the addition of an equal volume of 0.5 M Tris–HCl buffer (pH 8.0). After vigorous mixing, the upper water phase was removed and 0.1 M Tris–HCl (pH 8.0) was added. The same amount of chloroform/isoamyl alcohol (24:1 v/v) solution as the phenol was added to obtain the PCI solution (phenol: chloroform: isoamyl alcohol 25:24:1 v/v). Pyridine nucleotides extraction was performed using about 5 to 6-day-old main cultures corresponding to OD_730_ = 2‒4 (approx.) (Fig. S1). Before extraction, the cultures were maintained under darkness for an hour with air bubbling. After the dark adaptation, cells were harvested from the calculated volume of culture (2 ml / OD_730_) by centrifugation at 12,000 × g for 3 min. The cell pellet was suspended in 50 µL of 1 mM NaHCO_3_. For the extraction from dark-adapted cells, 300 µL PCI was added to the suspension followed by the addition of 250 µL extraction buffer (approx. pH 10.4‒11.0, production code: N509 or N510 Dojindo, Kumamoto Japan). For light-irradiated extraction, the cell suspension was transferred into a cuvette with 1 mm optical path to illuminate the cells. For actinic light, LED light source (pE-100wht, BioVision Technologies, Exton, PA, USA) was used through 550 nm long path filter. A 300-µL aliquot of PCI was added to the suspension in the cuvette at various time points, followed by the addition of 250 µL extraction buffer. The PCI suspension samples were centrifuged at 12,000 × g for 3 min. Each upper water phase was transferred to a microtube and frozen quickly in liquid nitrogen.

For extraction without PCI, only extraction buffer was added to the cells. The cell suspension was frozen and thawed once for cell disruption. Proteins in the cell lysate were removed by ultrafiltration before NADP(H) measurements. Other extraction processes were same as those for PCI.

### Enzymatic pyridine nucleotides measurement

For the PCI extraction samples, after thawing the pyridine nucleotides extraction, 250 µL chloroform/isoamyl alcohol (24:1 v/v) solution was added for further removal of phenol. The mixture was centrifuged at 12,000 × g for 5 min and the water layer was transferred into two microtubes for measurement of total pyridine nucleotides and reduced form of pyridine nucleotides (NADH and NADPH). The sample used for measuring reduced form of pyridine nucleotides was incubated at 60 °C for an hour to decompose oxidized form of pyridine nucleotides (NAD^+^ and NADP^+^) in the sample solution (NAD^+^ and NADP^+^ are unstable in heated alkaline solutions), while the sample used for measuring total pyridine nucleotides was kept on ice. For determination of the pyridine nucleotides concentration, the enzymatic cycling assay was performed according to the manufacturer’s instructions (production code: N509 for NAD(H); N510 for NADP(H), Dojindo Kumamoto Japan). The principle of the enzymatic measurements was summarized in a review paper (Kern et al. 2014). Absorbance at 450 nm of each sample in a 96-well plate was measured on Infinite M200 Plate Reader (Tecan, Männedorf, Switzerland).

## Results

### In vitro quantitation of NADP(H)

**Fig. 1:**
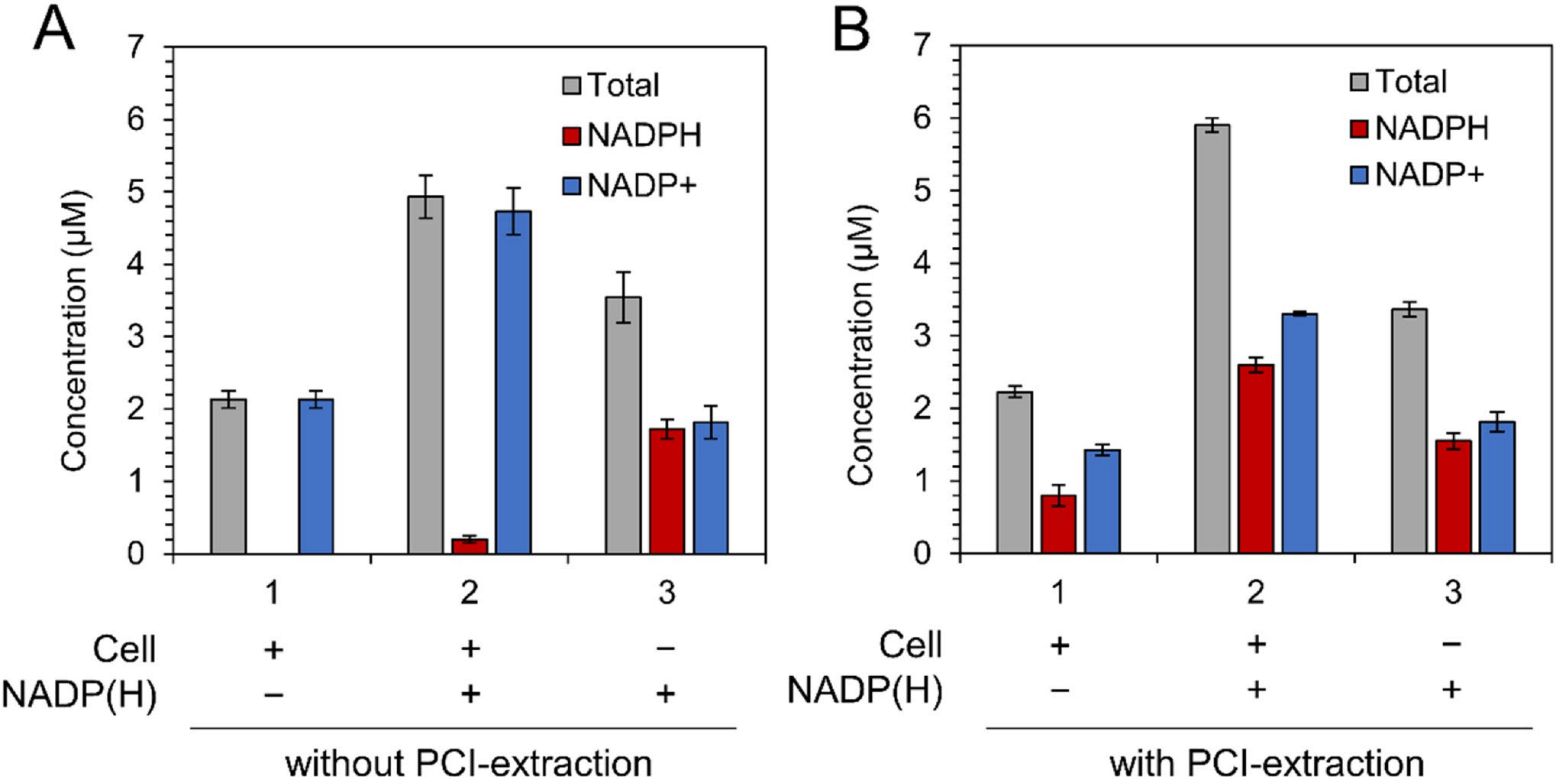
Effect of PCI solution on NADP(H) extraction. NADP(H) concentration in the crude extracts processed (**a**) without and (**b**) with PCI. Sample numbers are indicated on the horizontal axes; No. 1: crude extracts from dark-adapted cells, No. 2: crude extracts containing exogenous NADP(H), No. 3: exogenous NADP(H). Values are means ± SD (bars) of three biological replicates

**Fig. 2:**
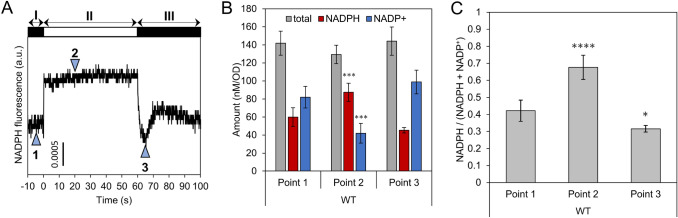
Light response of NADP(H) in wild type (WT). (**a**) NAD(P)H fluorescence transients affected by 1 min illumination. White and black bars indicate light and dark condition, respectively. Points indicated by triangles with numbers correspond to three different states; point 1: after 1 h dark adaptation, point 2: 20 s after light irradiation (200 μmol m^−2^ s^−1^), point 3: 5 s after transition to the dark condition. (**b**, **c**) NADP(H) contents and NADPH fraction against total NADP(H) content in WT were obtained by adding PCI to the cell suspension at each time point. The light intensity of 600 μmol m^−2^ s^−1^ was used. Values are means ± SD (bars) of about 3‒6 biological replicates. Significant differences from dark-adapted conditions were evaluated by a Student’s t test (*P < 0.05, ***P < 0.001, ****P < 0.0001)

**Fig. 3:**
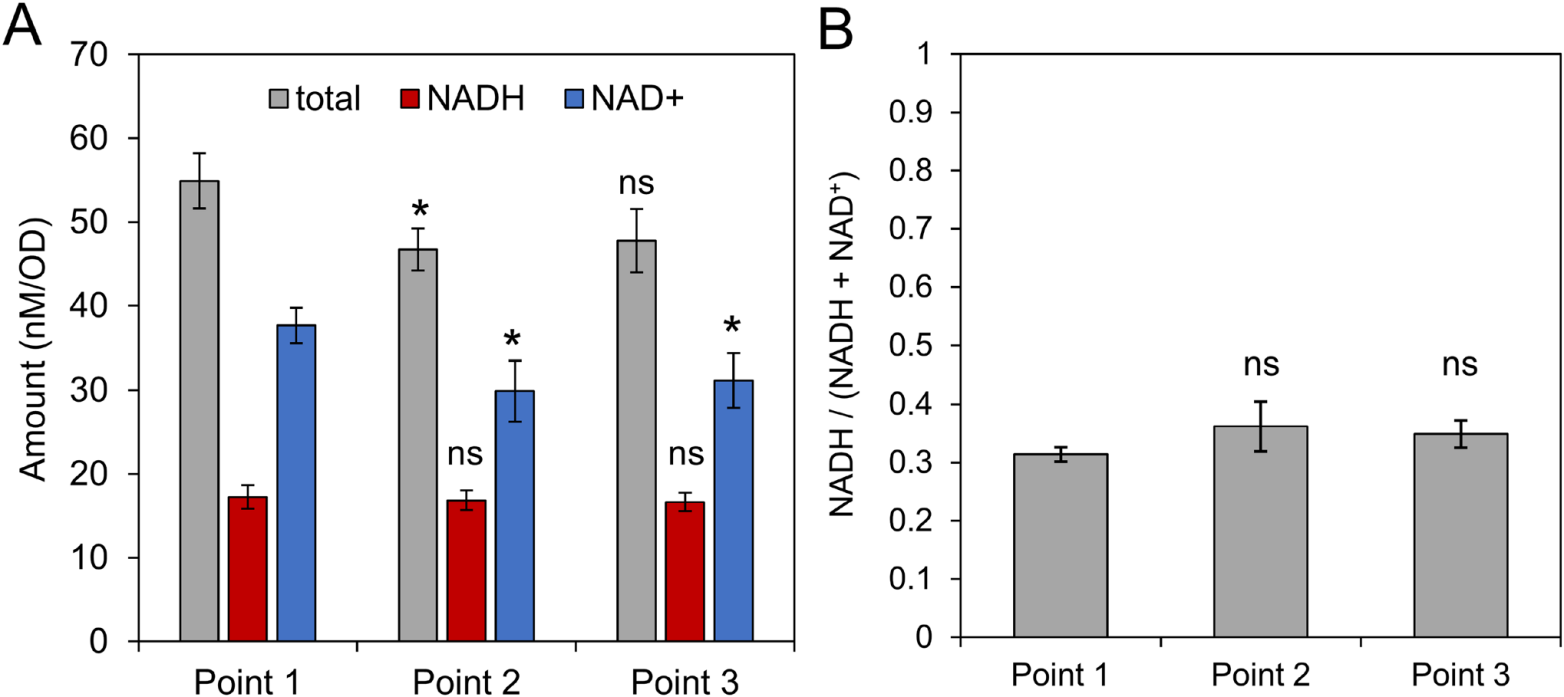
Effect of light irradiation on NAD(H) in wild type (WT). (**a**) NAD(H) contents and (**b**) NADH fraction against total NAD(H) content in WT were obtained by adding PCI to the cell suspension at each time point same as Fig. 2. The light intensity of 800 μmol m^−2^ s^−1^ was used. Values are means ± SD (bars) of 3 biological replicates. Significant differences from dark-adapted conditions were evaluated by a Student’s t test (ns: not significant; *P < 0.05)

First, NADP(H) was extracted from dark-adapted *Synechocystis* cells using extraction buffer, and subsequently quantified according to the standard protocol of the commercially available assay kit (see Materials and Methods for details). Although 2.1 µM of NADP^+^ in the extraction buffer was confirmed to be present, the reduced form, NADPH, was not detected (Fig. 1a–1). To verify the possibility that NADPH was unexpectedly oxidized during the extraction process, extraction was performed using a buffer containing exogenous 1.5 µM NADP^+^ and 1.5 µM NADPH as standards. The NADPH level was confirmed to be less than 1.5 µM even for this sample (Fig. 1a–2). Notably, when only the exogenous NADP^+^/NADPH mixture was examined, the concentrations were accurately quantified (Fig. 1a–3). These results clearly indicated that components in the cell lysate oxidized NADPH during the extraction process.

Considering that non-enzymatic degradation of NADP(H) is negligible due to the very slow kinetics, Fig. 1a suggests that the cell lysate contains enzymes that accelerate the redox reactions related to NADP(H) chemistry. To accurately quantify the intracellular NADP^+^/NADPH ratio, these proteins must be quickly inactivated during the extraction process. Therefore, NADP(H) was extracted in the presence of a PCI, a protein scavenging agent widely used for extracting nucleic acids (hereafter, this protocol is called PCI extraction). In this case, although the total concentration of NADP^+^ and NADPH was the same as that determined without PCI treatment, the NADPH value reached 0.8 µM (Fig. 1b–1). When 1.5 µM of exogenous NADP(H) was added following addition of PCI to the cells, the concentrations of NADPH and NADP^+^ both increased by approximately 1.5 µM (Fig. 1b–2). It was confirmed that PCI itself did not affect the measurement of exogenous NADP(H) (Fig. 1b–3) These results suggested that the intracellular concentrations of NADP(H) can be quantitatively determined using the developed PCI extraction protocol.

### Comparison of in vivo and in vitro measurements

To evaluate the validity of the PCI extraction protocol, the time-transient behavior of NADPH levels under varying irradiation conditions was compared with the results obtained using the NAD(P)H fluorescence measurement method. As shown in Fig. 2a, when the actinic light (200 μmol m^−2^ s^−1^) was turned on, the NAD(P)H fluorescence intensity rapidly increased (stage-II). When the light was turned off, the fluorescence intensity decreased and reached a minimum within 5 s, subsequently increasing and stabilizing after 30 s (stage-III). This time-transient behavior is in good agreement with previous reports, and the decrease and increase of the fluorescence level after turning the light off in stage-III are considered to be due to NADPH consumption by the Calvin cycle and NADPH production by the oxidative pentose phosphate pathway (OPPP), respectively (Mi et al. 2000; Kauny and Sétif 2014). The fluorescence-based method can detect NAD(P)H responses to environmental light changes on a time scale of seconds.

Next, the range of variation in the amount of NADP(H) was estimated using the PCI extraction method. PCI was added to cell suspension at three different points (Fig. 2a, points 1–3). Although the total amounts of NADP(H) were the same for all three samples, the NADPH fraction changed depending on the stage (Figs. 2b, c). The observed fraction of NADPH was 0.42 for the dark-adapted cells, which increased to 0.68 following light irradiation (600 μmol m^−2^ s^−1^), and then decreased to 0.32 at the onset of darkness. The time-transient behavior of NADPH levels under light/dark perturbations deduced using the PCI extraction protocol agrees well with that obtained using fluorescence measurements. However, although this light-dependent changes of fluorescence is generally considered to be originated from NADPH variations, there is a possibility that variation of NADH amount also affects the fluorescence behavior because the fluorescence measurement used in this study cannot distinguish fluorescence signal from NADPH and NADH. To verify light response of NADH, NAD(H) was also quantified by using PCI method. As a result, the amount and fraction of NADH was unchanged over the light–dark transition, indicating that time-transient changes of the fluorescence observed in this time scale was attributed to the variation of NADPH alone (Fig. 3).

**Fig. 4:**
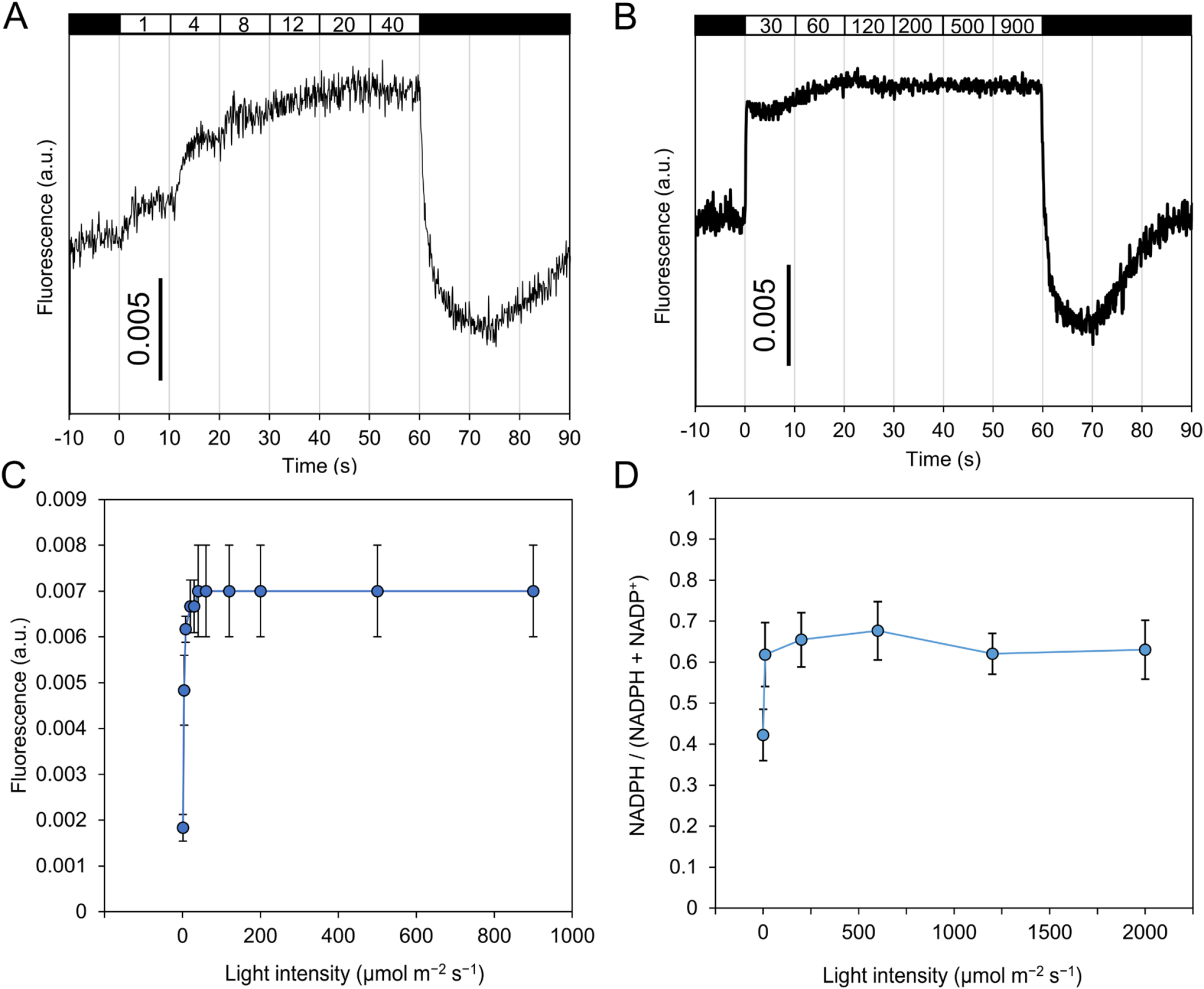
Effect of light intensity on NADPH reduction level. (**a**, **b**) NAD(P)H fluorescence trace measured under different light intensities. White and black bars indicate light and dark condition, respectively. The numbers in the white bars indicate light intensity (μmol m^−2^ s^−1^). Average traces of 3 biological replicate are shown. (**c**) Values of fluorescence signal at the end of each light intensity irradiation shown in (**a**) and (**b**) are plotted. Values are means ± SD (bars) of 3 biological replicates. (**d**) NADPH fraction under various light intensities measured by PCI extraction method. Values at 10, 200, 1200 and 2000 μmol m^−2^ s^−1^ are means ± SD (bars) of 4 biological replicates. Values at 0 and 600 μmol m^−2^ s^−1^ are identical to ones in Fig. 2c

The fraction of NADPH in light conditions (600 μmol m^−2^ s^−1^) was measured as 0.68 ± 0.07 in Fig. 2c, and it seems that there is a room for NADP(H) to be more reductive. To evaluate the maximum reduction level of NADPH, we investigated light intensity dependency of NADPH level by both fluorescence and PCI extraction methods. Interestingly, increasing light intensity more than 20 μmol m^−2^ s^−1^ did not give a significant rise on the fluorescence intensity, suggesting that redox state of NADP(H) was saturated at very weak light intensity (Figs. 4a–c). In fact, light intensity dependence of NADPH fraction measured by the PCI extraction method showed the similar saturation behavior (Fig. 4d). These results indicate that maximum photoreduced fraction of NADPH was 68%, and approximately 32% NADP(H) is kept oxidized even in light conditions by unknown mechanism(s) in this experimental condition.

**Fig. 5:**
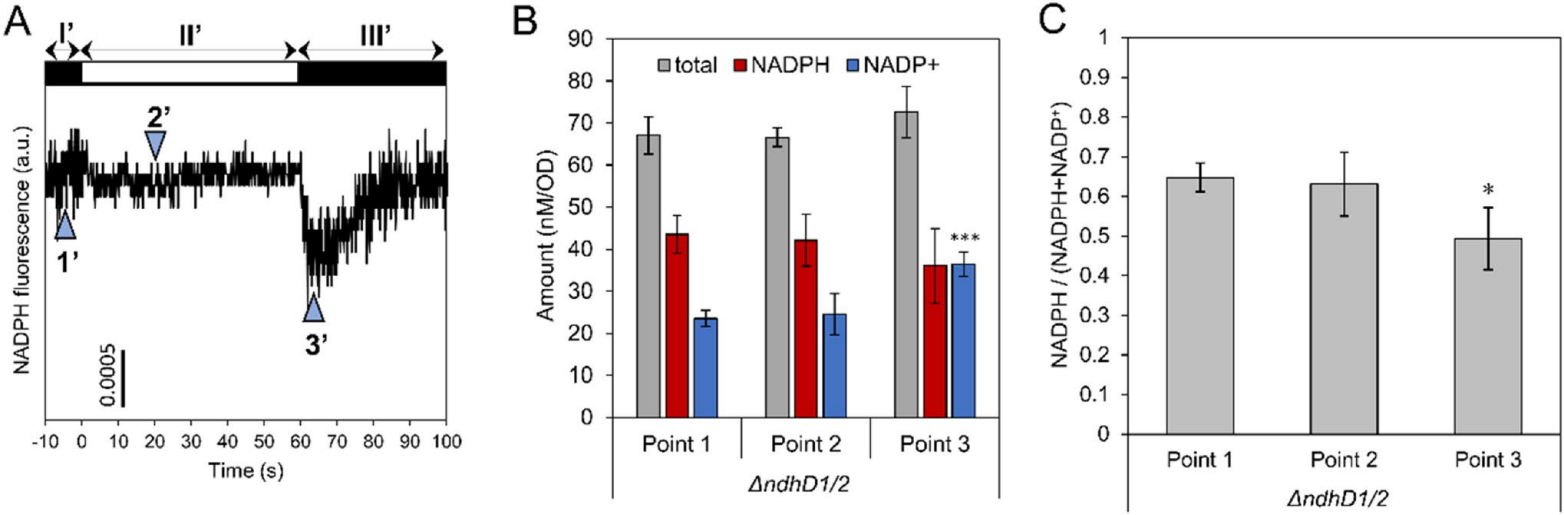
Light response of NADP(H) in *ΔndhD1/2* mutant. (**a**) NAD(P)H fluorescence transients affected by 1 min illumination. White and black bars indicate light and dark condition, respectively. Points indicated by triangles with numbers correspond to three different states; point 1: after 1 h dark adaptation, point 2: 20 s after light irradiation (200 μmol m^−2^ s^−1^), point 3: 3 s for *ΔndhD1/2* after transition to the dark condition. (**b**, **c**) NADP(H) contents and NADPH fraction against total NADP(H) content in *ΔndhD1/2* obtained by adding PCI to the cell suspension at each time point. The light intensity of 600 μmol m^−2^ s^−1^ was used. Values are means ± SD (bars) of 3 biological replicates. Significant differences from dark-adapted conditions were evaluated by a Student’s t test (*P < 0.05, ***P < 0.001)

The reliability of PCI extraction method was further verified using a mutant strain lacking *ndhD1* and *ndhD2* (*ΔndhD1/2*). In this mutant strain, which lacks the ability to oxidize NAD(P)H in the respiratory chain through the Type I NAD(P)H dehydrogenase complex (NDH-1) (Ohkawa et al. 2000), the relative ratio of NADPH at the dark-adapted state is considered to be as high as that in the light-adapted state (Sétif et al. 2020). In fact, NAD(P)H fluorescence levels did not change when the light was turned on (Fig. 5a, stages I’ and II’). Although the fluorescence level decreased transiently after the light was turned off, it quickly recovered to the same level as in the light condition (stage-III’). This behavior was consistent with previous results (Sétif et al. 2020). The PCI extraction method was then applied to the mutant strain for comparison with the results of NAD(P)H fluorescence measurements. For the mutant strain, there was no significant difference in the NADPH fraction between the dark-adapted and light-irradiated points (Figs. 5b, c, points 1′ and 2′), whereas a decrease in the NADPH fraction was observed 3 s after the light was turned off (point 3′). This behavior is consistent with that obtained by the NAD(P)H fluorescence.

The amount of light-dependent NADPH (difference between points 1 and 2 in Fig. 2a) was overestimated in the fluorescence method. This is because of that the fluorescence yield from intracellular protein-bound NADPH is generally higher than the fluorescence yield from extracellular free NADPH (Latouche et al. 2000). Since the calibration curve was obtained using extracellular free NADPH, the gap between intracellular and extracellular fluorescence yields needs to be corrected. Kauny and Sétif reported that in vivo NAD(P)H fluorescence was estimated to be 2–4 times (‘the enhancement factor’) higher than the exogenous NADPH fluorescence (Kauny and Sétif 2014). In our results, light-dependent NADPH was estimated to be 20.9 and 9.0 nmol mg^−1^ Chl using the fluorescence-based and PCI extraction methods, respectively (Fig. 2 and S2). Thus, the enhancement factor was 2.3, which is within the range reported by Kauny and Sétif, supporting the reliability of the PCI extraction method.

## Discussion

In photosynthetic organisms, the photochemical reaction causes subcellular dynamic changes of redox state. Considering that Calvin cycle enzymes including GAPDH are arranged peripheral to thylakoid membrane in chloroplasts of higher plants and green algae (Suss et al. 1993, 1995), the diffusion length of photogenerated NADPH would influence on photosynthetic efficiency. Therefore, clarifying the diffusion and localization of photoreduced NADPH in stromal space of cyanobacteria and chloroplasts is important for understanding the photosynthetic reactions. Recent studies using fluorescent protein sensors revealed that the dynamic changes in NAD(P)H are organelle-specific in *Arabidopsis thaliana* (Lim et al. 2020). On the other hand, dynamics of local redox state within chloroplasts or cyanobacteria (with the size of sub-micrometers), where photosynthetic electron transfer reaction directly changes redox states, is still largely unknown. In the light of this situation, the quantitative information on the redox ratio of NADP(H) obtained in this study will provide novel insights into the redox dynamics as described below.

Our experimental results clearly show that a fraction of the NADP(H) pool is kept oxidized even in light conditions. Since NADP^+^ is an essential co-factor for G6PDH, 6PGDH, ICD and succinic semialdehyde dehydrogenase (Muro-Pastor and Florencio 1992; Zhang and Bryant. 2011; Ishikawa and Kawai-Yamada 2019; Ito and Osanai. 2020), which are components of primary metabolism pathways in *Synechocystis*, some portion of NADP^+^ pool needs to be stably present even under light conditions. On the other hand, NADPH provides essential reducing power for maintaining antioxidant ability through enzymes such as NTR and GR under both light and dark conditions (Hishiya et al. 2008; Yoshida and Hisabori 2016; Vogelsang and Dietz 2020). Thus, given that NADP^+^ and NADPH have their own distinctive roles in photosynthetic cells, maintaining an appropriate redox balance of NADP(H) pool is expected to be important in facilitating various biological processes cooperatively in fluctuating light environments. For plastoquinone (PQ), one of the major intracellular redox species, it is reported that photoactive and non-photoactive pools exist in both chloroplasts and cyanobacteria (Kruk and Karpinski 2006; Khorobrykh et al. 2020). In fact, we found that non-photoactive PQ accounted for 87% of the PQ pool in *Synechococcus elongatus* PCC 7942 cells (Fig. S3). Although NADP(H) is located in the cytoplasm, and cyanobacteria lack organelles, some portion of NADP(H) can be kept oxidized even in light conditions in a cyanobacterial cells by an unknown mechanism. For example, it was reported that NADPH allosterically decreases the binding affinity of ferredoxin (Fd) to ferredoxin-NADP^+^ reductase (FNR) (Kimata-Ariga et al. 2019), which may prevent complete reduction of the NADP^+^ pool. Another possible explanation is that NADP(H) non-specifically binds proteins such as Rubisco (Badger and Lorimer 1981; Latouche et al. 2000), which may represent the bulk of the less active NADP(H) pool. Furthermore, the possibility that some proportion of NADP(H) is spatially sequestered into unknown compartments in cyanobacterial cells cannot be ruled out. Thus, there is room for further investigation toward unveiling the nature of the NADP(H) pool.

Next, let us consider the reasons for the increase and decrease of the NAD(P)H fluorescence. It is obvious that NADPH is increased by the reduction of NADP^+^ at the PETC under illumination. The initial decay in NAD(P)H fluorescence just after the onset of the dark phase (Fig. 2a, before point 3 in stage-III) can be explained by the increased consumption of NADPH in the Calvin cycle (Kauny and Sétif 2014). The subsequent increase in fluorescence intensity (after point 3) is thought to be due to NADPH production by increased OPPP activity. In addition, the *ΔndhD1/2* strain exhibited different time-transient behavior in fluorescence intensity from that observed for the WT (Fig. 5a), indicating that not only the PETC but the respiratory electron transport chain can also lead to light-responsive change in NADP(H) amount. Moreover, as shown in Fig. 5b, the total amount of NADP^+^ and NADPH for the mutant was approximately half of that for the WT. On the other hand, since NADH also exhibit fluorescence, it is necessary to verify whether NADH variation also affects typical time transients of NAD(P)H fluorescence shown in Fig. 2a and reported in previous papers (Mi et al. 2000; Kauny and Sétif 2014; Holland et al. 2015; Shaku et al. 2016). In fact, a recent study for chloroplasts *in planta* showed that NADH also increased by light irradiation (Lim et al. 2020), raising the above possibility. However, we found that NADH in *Synechocystis* was hardly changed over the light irradiation for at least one minute (Fig. 3). Taken together, variation of NADPH amount due to activities in the photosynthetic and respiratory electron transport chains, Calvin cycle, and OPPP contributes to the time-transient changes of in vivo NAD(P)H fluorescence at least within one minute.

As described, we have successfully determined the absolute amount of NADP(H) and the NADPH fraction using PCI during the extraction process. The total NADP(H) amount in *Synechocystis* was estimated as 34.3 ± 3.4 nmol mg^−1^ Chl. This value is close to that in chloroplasts of plant protoplasts with high CO_2_ concentration (19.5 ± 1.7 nmol mg^−1^ Chl, Wigge et al. 1993) and in intact chloroplasts of spinach (approximately 40 nmol mg^−1^ Chl, Lendzian and Bassham 1975). The results obtained using this in vitro method were in very good agreement with the results obtained from the in vivo fluorescence method, demonstrating that this treatment is capable of halting cellular redox within at least a few seconds. This capability is likely to be attributed to the quick inactivation of proteins by phenol, which prohibits further changes in the redox state of NADP(H). Importantly, our quantitative analyses revealed that the some portion of NADP(H) pool is maintained in oxidized form even in light conditions. In contrast to the PCI extraction method, the methods based on fluorescence protein sensors enable in vivo imaging of redox state. However, since the fluorescence yield is easily influenced by various environmental factors such as local pH, the quantitative estimation of the absolute concentration and redox ratio is, in general, technically difficult (Lim et al. 2020; Sugiura et al. 2020). Thus, there is a complementary relationship between the fluorescent-based in vivo method and our quantitative in vitro method. We anticipate our novel protocol for NADP(H) quantification established in the present work will deepen our understanding of the regulation mechanisms and physiology of photosynthesis.

## Supplementary Information

Below is the link to the electronic supplementary material.

Supplementary file1 (DOCX 779 kb)
