# Supplementary material for: Quantification of NAD(P)H in cyanobacterial cells by a phenol extraction method
Source: Photosynth Res. 2021 May 2;148(1):57–66. doi: 10.1007/s11120-021-00835-1 (PMC8154815; doi:10.1007/s11120-021-00835-1)
Supplement: Supplementary file 1 — Supplementary file1 (DOCX 779 kb) [file 11120_2021_835_MOESM1_ESM.docx]

**Supplementary Information for**

A quantification of NADP^+^/NADPH redox ratio by phenol extraction method in cyanobacterial cells

*Kenya Tanaka^1^, Ginga Shimakawa^2^, Hiro Tabata^1^, Shoko Kusama^1^, Chikahiro Miyake^3^ and Shuji Nakanishi^1, 2,*^*

*Corresponding author: *Shuji Nakanishi*.

Email: nakanishi@chem.es.osaka-u.ac.jp

**Supplementary Information Text**

**Measurement of plastoquinone.** Redox ratio of plastoquinone (PQ)/ plastoquinol (PQH_2_) was determined by the method described in Khorobrykh et al., 2020. Briefly, *Synechococcus* *elongatus* PCC 7942 cells harvested by vacuum filtration from calculated volume of culture (4 mL /OD_730_) were grinded in 2 mL ice-cold ethyl acetate. 300 μL of the filtered cell lysate was dried with N_2_ gas. The obtained pellet was dissolved in 300 μL methanol. The PQH_2_ amount was determined by fluorescence intensity at 330 nm excited at 290 nm in HPLC. To measure total PQ amount, all PQ in a portion of the sample was reduced to PQH_2_ by addition of NaBH_4_ at the final concentration of 62.5 μM.

**SI References**

Khorobrykh S, Tsurumaki T, Tanaka K, Tyystjärvi T, Tyystjärvi E (2020) Measurement of the redox state of the plastoquinone pool in cyanobacteria. FEBS Lett 594: 367-375

**
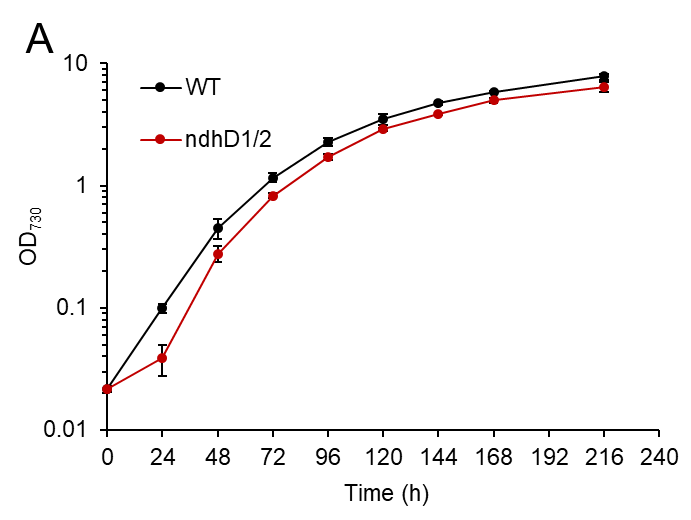

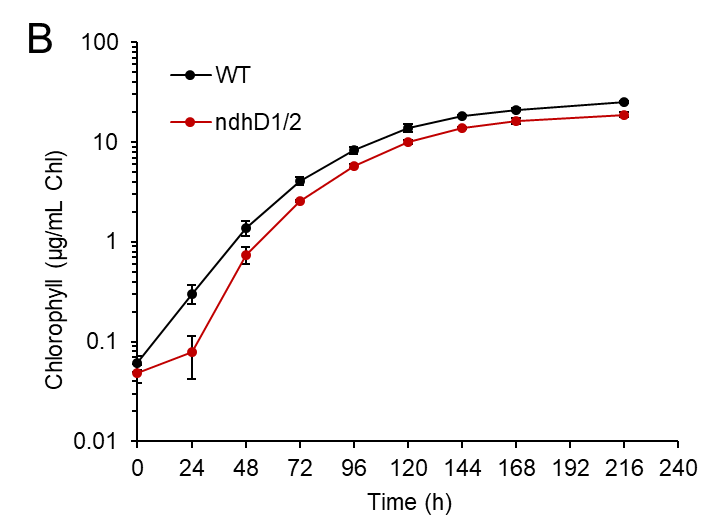

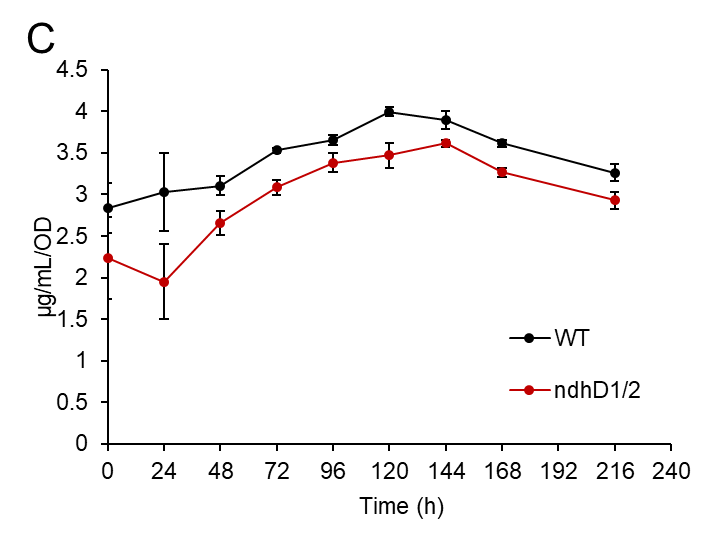
**

**Fig. S1.** Growth curves of *Synechocystis* strains used in this study. Time course of (A) optical density at 730 nm (OD_730_) and (B) chlorophyll concentration were measured for cultures grown under constant light conditions. (C) Chlorophyll concentration per OD_730_ was calculated for unit conversion of NADP(H) concentration (See Table 1). Values are means ± SD (bars) of three biological replicates.


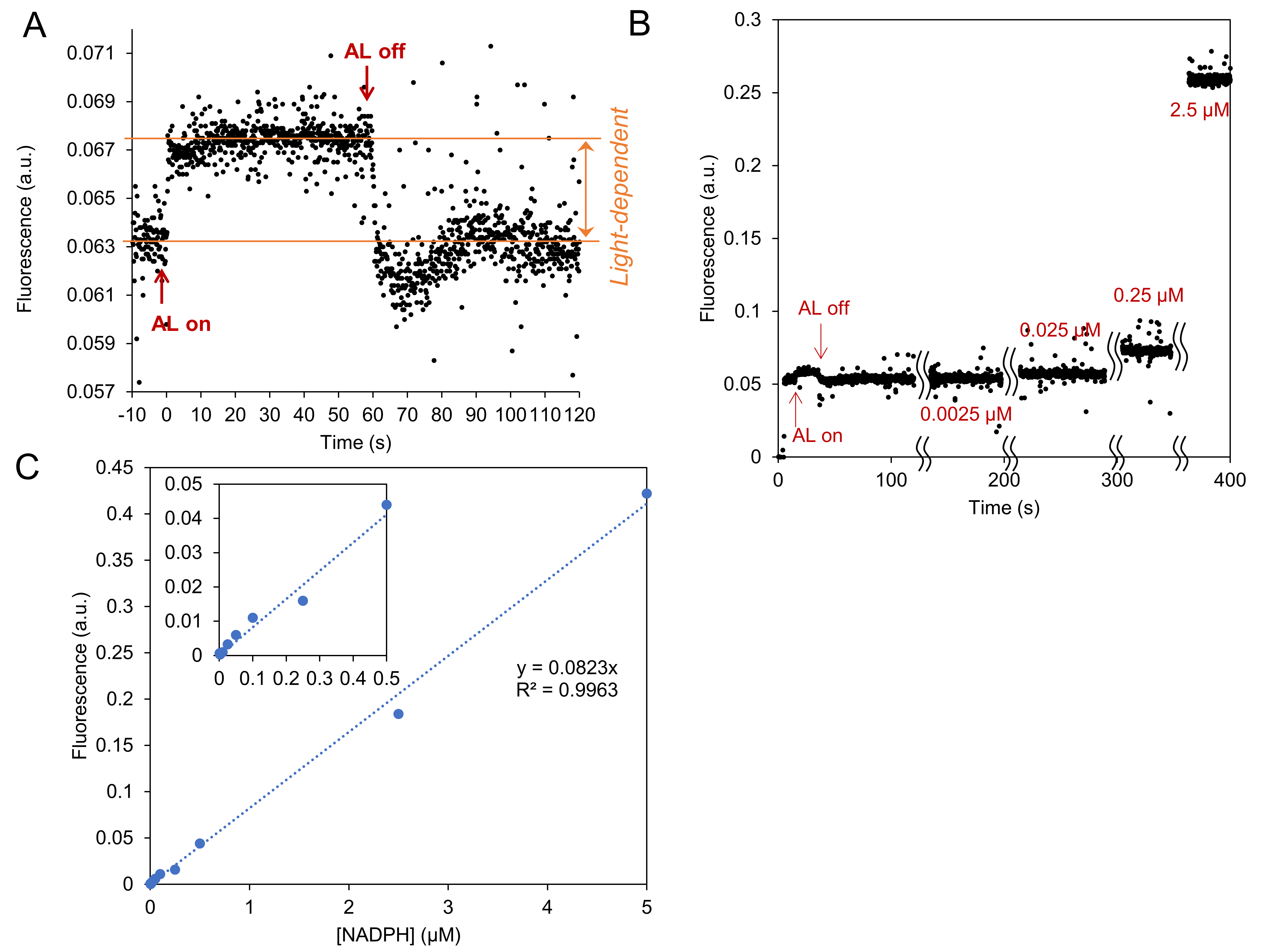


**Fig. S2.** (A) Light-dependent NAD(P)H fluorescence changes in WT cells. Irradiation of actinic light (AL) was started or stopped at the indicated arrows. (B) Correlation between NADPH concentration and fluorescence intensity was verified by adding known amount of NADPH into cell suspension containing 2.5 μg Chl ml^-1^ of wild type cells, same concentration as measurement for light response. Concentration of exogenous NADPH is indicated along the trace. Representative fluorescence trace of 3 replicates is shown. (C) Calibration curve for conversion from NAD(P)H fluorescence intensity to absolute NADPH amount. NAD(P)H fluorescence increase shown in (B) was plotted against known concentration of exogenous NADPH. Inset shows the enlarged view of the graph. Light-dependent NADPH amount was estimated as 20.9 ± 2.4 nmol mg^-1^ Chl. The value is means ± SD of three technical replicates.


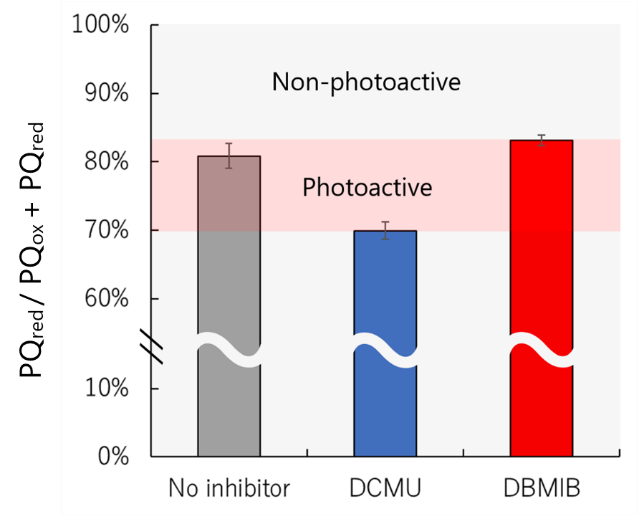


**Fig. S3.** Photoactive and non-photoactive plastoquinone (PQ) ratio in *Synechococcus* *elongatus* PCC 7942 cells. Photo-active PQ ratio was determined as the difference in PQ redox ratio between DCMU-added sample and DBMIB-added sample. Values are means ± SD (bars) of three independent experiments.
